# Clonal hematopoiesis JAKs up plaque formation

**DOI:** 10.1172/JCI187529

**Published:** 2025-01-02

**Authors:** Koral Campbell, Qing Li

**Affiliations:** 1 Department of Pathology,; 2 Department of Medicine, Division of Hematology/Oncology, and; 3 Department of Cell and Developmental Biology, University of Michigan, Ann Arbor, Michigan, USA.

## Abstract

Clonal hematopoiesis (CH) is a condition in which hematopoietic stem cells (HSCs) acquire mutations seen in leukemia. While individuals with CH generally do not show signs of hematologic disease, the condition becomes more common with age and correlates with age-related diseases, especially cardiovascular disease (CVD). *JAK2* mutations in HSCs can lead to CH and correlate with atherosclerosis, but the condition has been difficult to study because of challenges modeling the mutant cells at very low frequency. In this issue of the *JCI*, Liu et al. developed a low-allele-burden (LAB) mouse model in which a small number of bone marrow cells carrying the *Jak2*^VF^ mutation were transplanted into mice predisposed to hyperlipidemia. Along with recapitulating features of plaque development, the authors identified the phagocytic receptors MERTK and TREM2 in WT cells as downstream of the inflammatory cytokine IL-1. These findings provide potential targets for preventing or treating patients at risk for CH-associated CVD.

## The association between clonal hematopoiesis and cardiovascular disease

Clonal hematopoiesis (CH) is a condition in which aged hematopoietic stem cells (HSCs) acquire somatic mutations commonly seen in leukemia despite no overt signs of hematologic disease. CH is a premalignant condition characterized by a mutation with a variant allele frequency (VAF) of at least 2% and can be detected in approximately 10% of individuals over the age of 65. The risk of acquiring a CH mutation increases by approximately 6% every 10 years (1, 2), and the overall survival rate of individuals with CH is reduced when compared with those without a CH mutation (3). Approximately 20 mutations have been classified as CH mutations, with *DNMT3A*, *TET2*, and *ASXL1* being the three most common; however, other genes coding for splicing factors or signaling pathway genes, such as *JAK2*, are also commonly detected (2, 3).

There is increasing evidence that CH is associated with many age-related nonhematologic diseases. One plausible explanation is that the increased cytokine secretion from the immune cells that carry the CH mutations contributes to many disease conditions. Recent work has shown that CH is associated with an increased risk for acute kidney injury (4), liver fibrosis (5), diabetes and insulin resistance (6), and autoimmune conditions, such as rheumatoid arthritis (7). While CH is linked to a higher incidence of hematologic malignancies, most CH-related mortality is attributed to cardiovascular disease (CVD) (2). There have been numerous studies investigating the link between clonal hematopoiesis and cardiovascular conditions, including myocardial infarction, atherosclerosis, and ischemic stroke (8). Studies using mouse models to investigate the contribution of mutant CH cells to nonhematologic conditions were mostly conducted in the *Tet2*-KO mice. These studies suggest that the contribution of CH clones to atherosclerosis development could result from a myeloid bias in which mutated HSCs produce increased quantities of monocytes and macrophages that promote IL-1, IL-6, and TNF production and drive systemic and vascular inflammation (9, 10). Although *JAK2* mutations are associated with a 12-fold increase in CVD risk compared with other CH mutations such as of *TET2* (9), the mechanisms by which *JAK2* mutations in blood cells promote plaque formation and thrombosis have not been fully investigated. The limited research conducted in *Jak2-*mutant mouse models has uncovered intriguing roles of immune cells, like macrophages, in CVD and has examined the connection between CVD and myeloproliferative neoplasms (MPNs) induced by *Jak2* mutations (10–12).

*JAK2* encodes a protein tyrosine kinase and is a critical component of the JAK/STAT pathway, which is activated by cytokines and growth factors. When a cytokine binds to its receptor, JAK2 becomes activated and, in turn, activates STAT proteins and many other pathways including PI3K/AKT and MAPK signaling. *JAK2* mutations such as *JAK2^V617F^* (also known as *JAK2^VF^*) result in constitutively active JAK2 kinase and the downstream signaling pathways. *JAK2* mutations are common in MPNs, such as polycythemia vera (PV) (approximately 90%–95%), essential thrombocytopenia (ET) (60%), and primary myelofibrosis (PMF) (approximately 57%) (13–16). The role of mutant *JAK2* in hematopoiesis has been studied in mouse models utilizing the knockin (KI) strategy of *Jak2^VF^*, the most common *JAK2* mutation in CH and myeloid neoplasms. Interestingly, most *Jak2-*KI mice die of thrombotic events (17), highlighting the strong proinflammatory signaling activated by hyperactive *Jak2* mutations. One potential issue of using this model to study CH is the near-complete replacement of WT bone marrow cells with the *Jak2^VF^*-mutant cells, which does not recapitulate CH, as most cases harbor mutations at a very low VAF. A previous study using a chimeric transplant model reported that *Jak2^VF^*-mutant bone marrow cells promote plaque development (18), but the VAF used in that study, at 20%, was much higher than the VAF commonly observed in individuals with CH, which is typically under 10%.

## An alternative approach

In the current issue, Liu et al. (19) reported the effects of *Jak2^VF^-*mutant bone marrow cells on atherosclerosis development using a low-VAF model. The authors developed a low-allele-burden (LAB) transplant model by transplanting 1.5% *Jak2^VF^*-mutated bone marrow combined with 98.5% GFP-labeled WT bone marrow into lethally irradiated hyperlipidemic *Ldl^–/–^* mice (19). This model has several advantages. (a) Because most CH mutations exhibit a low VAF, the LAB model provides a more clinically relevant approach for studying the effects of CH on atherosclerosis. (b) The use of the GFP-labeled WT bone marrow cells allowed the authors to evaluate the contribution of WT and mutant cells and their crosstalk. (c) By utilizing the *Ldl^–/–^* mice as transplant recipients and feeding them a Western diet (WD), the authors were able to create the high-lipid and cholesterol environment that is important for plaque development. (d) Finally, the LAB model showed normal blood cell counts and spleen size, with no expansion of *Jak2^VF^* alleles within WBCs, neutrophils, monocytes, or monocyte subsets, therefore mimicking clinical CH. This allows for the observation of atherosclerotic development without effect of other common secondary conditions, such as MPN or leukemia. Although a nontransplant LAB mouse model would be preferable to avoid the effects of irradiation, a consistent, tissue-specific low-VAF model has yet to be developed (20). Together, the model developed by Liu et al. (19) provides an excellent method to study clinically relevant levels of CH.

**Figure 1:**
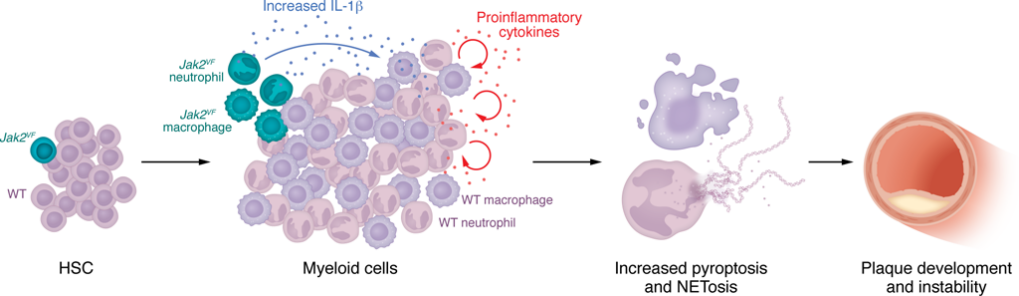
Crosstalk between sparse *Jak2-*mutant CH clones and WT myeloid cells promotes plaque formation. Sparse *Jak2^VF^*-mutant HSCs generate myeloid cells that release high levels of IL-1, which is received by the WT macrophages and neutrophils. In this context, IL-1 induces inflammasome signaling and, eventually, pyroptosis and NETosis, increasing the recruitment of immune cells to the plaques and the size of the necrotic core, which promotes plaque instability.

This LAB model allowed the authors to address a key question: How does a very low number of mutant blood cells promote a proinflammatory condition that leads to atherosclerosis? By transplanting 1.5% *Jak2^VF^* cells with 98.5% GFP^+^ bone marrow into *Lld^–/–^* mice, the authors demonstrated that even a low level of CH cells was sufficient to drive plaque development (19). They also found that JAK2 WT bone marrow cells cotransplanted into *Lld^–/–^* mice contributed to atherosclerosis in an IL-1R–dependent manner. Previous work has shown that IL-1 signals mediate crosstalk between immune cells and stromal cells, promoting aging in bone marrow (21). The current study (19) implies that the crosstalk is much more extensive, involving many different cell types. Given that the CH clone produces elevated IL-1β levels, Liu and co-authors propose a model of IL-1β/IL-1R signaling–mediated crosstalk between CH and WT myeloid cells (Figure 1). IL-1β from CH cells acted on healthy myeloid cells, triggering inflammasome-induced pyroptosis in macrophages and NETosis in neutrophils, two of the critical processes in atherosclerotic progression and instability (22, 23). Deletion of IL-1R in JAK2 WT bone marrow cells reduced NETosis, pyroptosis, vascular plaque size, and necrotic core size, underscoring the role of IL-1β–IL-1R crosstalk in driving plaque development and instability.

## Clinical implications

These results emphasize the potential for IL-1 inhibitors in the treatment of CVD. In a recent clinical trial, canakinumab, an FDA-approved IL-1 inhibitor for the treatment of rheumatoid arthritis, has shown promising results in patients carrying CH driver *TET2* mutations and a history of myocardial infarction (24, 25). This finding indicates that interrupting the chronic inflammation caused by the IL-1 cytokine cascade is important for stalling atherosclerosis development and progression. However, while canakinumab and other IL-1 inhibitors have shown promising results in patients with CVD, they are also associated with an increased risk of infection, making it important to develop alternative targeted therapies. Liu et al. identified two downstream pathways of IL-1 — MERTK and TREM2 — as important mediators of its effects on atherosclerotic development in mice (26, 27).

MERTK is a receptor tyrosine kinase that plays an important role in efferocytosis. Previous studies showed that deletion of *Mertk* increases necrotic core size and plaque instability (26). In the current study, Liu et al. (19) transplanted *Jak2^VF^* cells along with bone marrow cells expressing hyperactive *Mertk (Mertk^CR^*) into the LAB mice prone to develop atherosclerosis. The mice showed improved necrotic cores and fibrotic caps and reduced neutrophil extracellular traps (NETs) compared with *Jak2^VF^* mice with WT *Mertk* (19). Additionally, the authors also investigated *Trem2* as a possible downstream target. *Trem2*-KO mice have shown expedited atherosclerosis development, possibly due to disruption of the balance between foam cell death and the clearance of necrotic cells (27). Here, Liu et al. (19) showed that a *Trem2* agonistic antibody, 4D9, induced an increase in fibrotic cap size, stabilizing the plaques. They proposed that this outcome was due to an increase in *Trem2^+^*PDGFB^+^ macrophages and PDGF receptor-α^+^ fibroblast–like cells in the caps (19). While the mechanism behind these pathways and how they act in coordination needs to be further investigated, these results suggest that targeting downstream pathways may provide a promising alternative to IL-1 inhibitors to mitigateatherosclerosis development.

Liu et al. present an interesting LAB model that offers a more clinically relevant system of investigating the role of CH in CVD. This study further highlights the role of WT myeloid cells in plaque formation in the presence of the small *Jak2^VF^* CH clones. The identification of IL-1 and its downstream targets, MERTK and TREM2, as important mediators of these effects provides further proof of concept that breaking the crosstalk between CH mutant and WT bone marrow cells represents a potential strategy to prevent or treat CVD in patients with CH.

## Version 1 01/02/2025

Electronic publication
